# Supplementary material for: Characterization of the Interaction Between SARS-CoV-2 Membrane Protein (M) and Proliferating Cell Nuclear Antigen (PCNA) as a Potential Therapeutic Target
Source: Front Cell Infect Microbiol. 2022 May 23;12:849017. doi: 10.3389/fcimb.2022.849017 (PMC9168989; doi:10.3389/fcimb.2022.849017)
Supplement: Supplementary file 1 [file DataSheet_1.pdf]

Supplementary Material

1. Supplementary Figures.

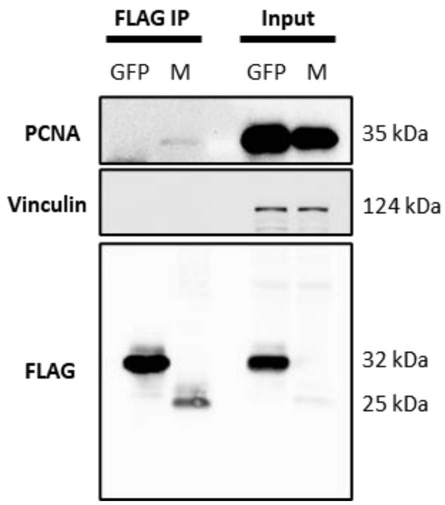

Supplementary Figure 1. Confirmation of M interaction with PCNA in another immunoprecipitation assay.

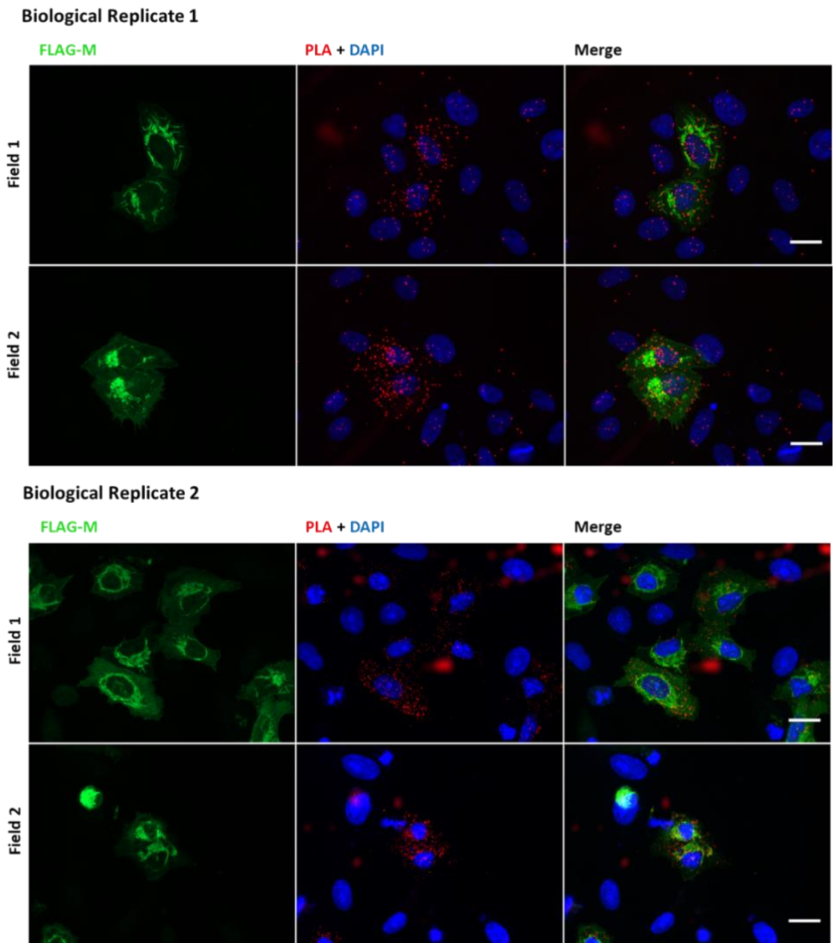

Supplementary Figure 2. Representative fields of PLA positive signal. The figure shows two different fields of non-cropped images for two independent biological replicates. All images were taken at 63× magnification with a ZEISS Axio Vert.A1 microscope. Scale bars 20 µm

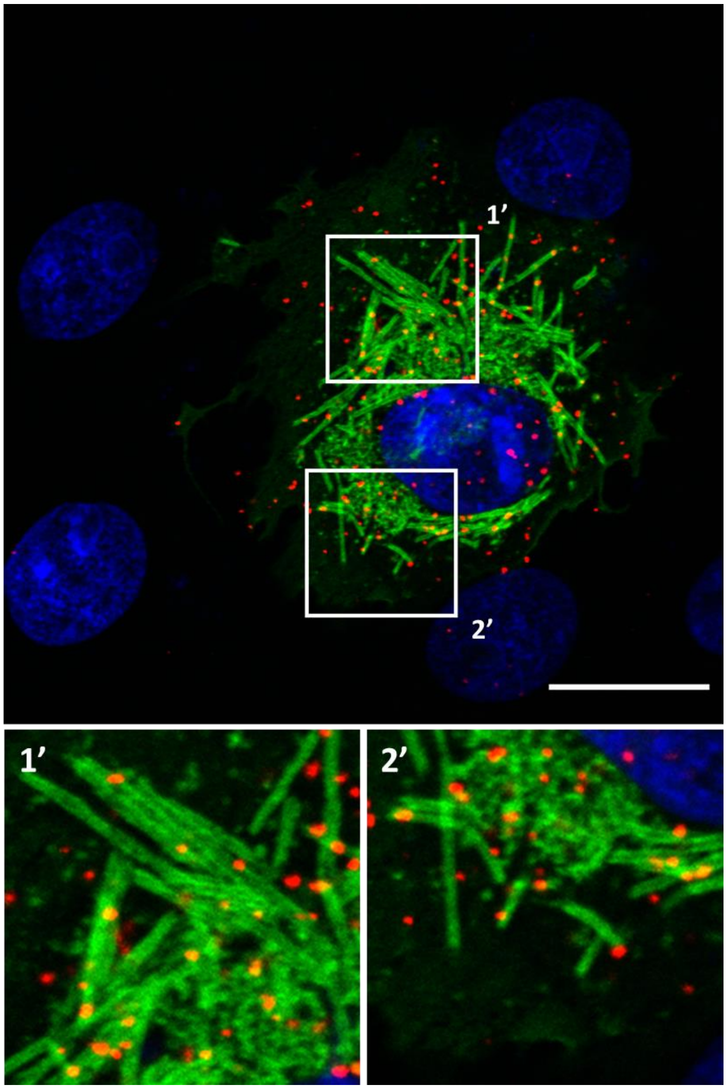

Supplementary Figure 3. Detection of FLAG-M/PCNA interaction by proximity ligation assay. Positive PLA cells were analyzed by confocal immunofluorescence to investigate proximity between PLA signal and FLAG-M membranous structures. Panels 1’ and 2’ represent zoomed areas indicated by framed regions in merge panel, showing PLA dots colocalizing with FLAG-M. The figure shows a single plane from a z-stacked image. Images were taken at 100× magnification with a Zeiss LSM-780-NLO microscope. Scale bars 20 µm. Detection of FLAG-M/PCNA interaction by proximity ligation assay.

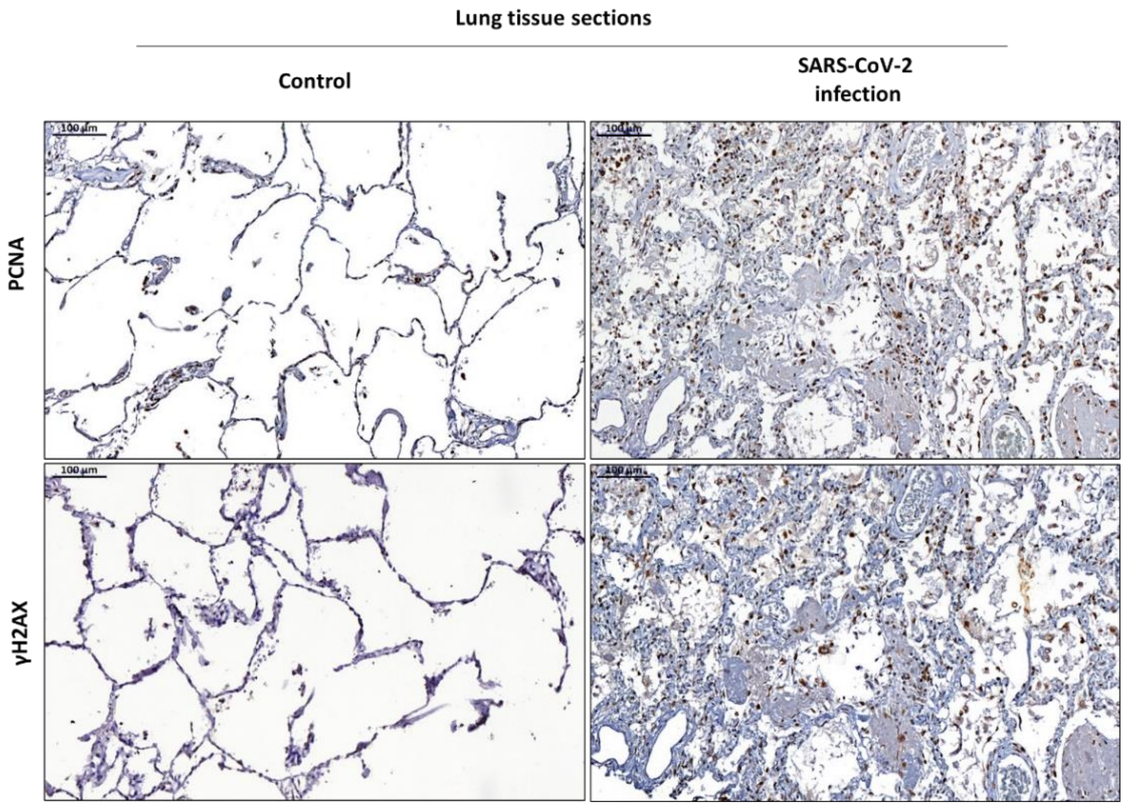

Supplementary Figure 4. Immunohistochemistry of lung sections from one control case and one COVID-19 patient immunostained with PCNA and γH2AX. Immunohistochemical positivity (brown color) for PCNA and γH2AX proteins are present in cells along the alveolar epithelium, with a higher density of positively stained cells for both markers in the COVID-19 case (right panels). This data is representative of one independent experiment.Confirmation of M interaction with PCNA in another immunoprecipitation assay.

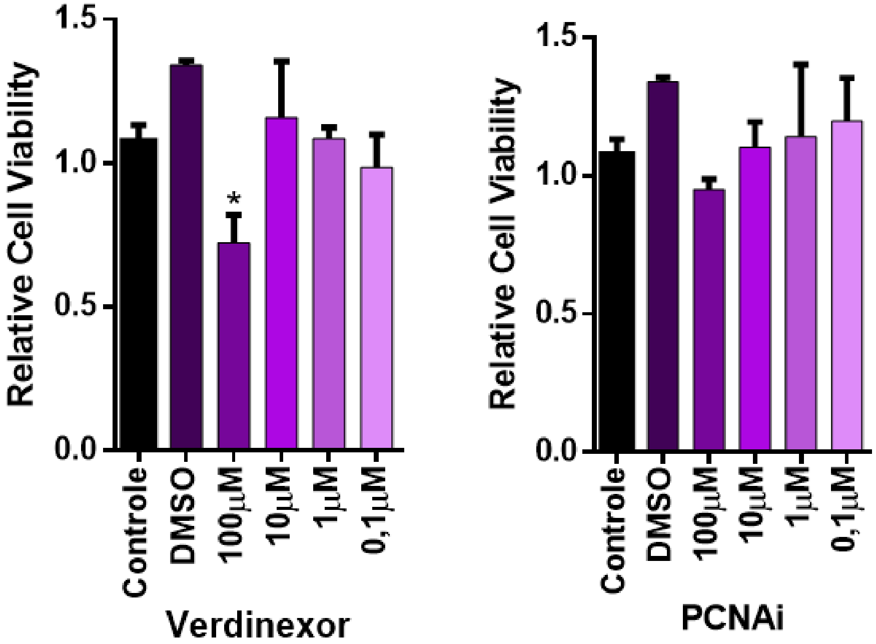

Supplementary Figure 5. Cell viability of VERO E6 after the treatment with different doses of Verdinexor and PCNAi. A MTT assay demonstrating that a dose of 100 µM affected the viability of the cells, but the doses of Verdinexor 1 and 0.1 µM and PCNAi 0.5 and 0.1 µM did not.
